# Bronchial Thermoplasty in Severe Asthma: Bridging Randomised and Real-World Evidence: A Systematic Review and Meta-Analysis

**DOI:** 10.3390/arm94050077

**Published:** 2026-10-21

**Authors:** Maria João Lúcio, Maria Luiza Pereira, Catarina Barata, Carlos Lopes, Jafar Aljazeeri

**Affiliations:** 1 Pulmonology Department, Hospital Egas Moniz, Unidade Local de Saúde Lisboa Ocidental, 1349-019 Lisboa, Portugal; catarinabarata22@gmail.com; 2 Faculty of Medicine, Universidade Nove de Julho, São Paulo 01525-000, Brazil; mluizappereira@gmail.com; 3 Severe Asthma Medical Unit, Unidade Local de Saúde Santa Maria, 1649-035 Lisboa, Portugal; carlosjlopes96@gmail.com; 4 Instituto de Saúde Ambiental (ISAMB), Faculty of Medicine, University of Lisbon, 1649-028 Lisboa, Portugal; 5 Department of Medicine, Medical City Hospital for Military and Security Services, P.O. Box 721, Muscat 111, Oman; jafar.aljazeeri@medportal.ca; 6 Medical Center, University of Pittsburgh, Pittsburgh, PA 15213, USA; 7 College of Medicine, Drexel University, Philadelphia, PA 19129, USA

**Keywords:** bronchial thermoplasty, biologic, exacerbations, quality of life, asthma control

## Abstract

**Highlights:**

**What are the main findings?**

- In randomised trials, bronchial thermoplasty improved asthma-related quality of life, asthma control, and morning peak expiratory flow. These improvements were observed mainly in trials without a sham control. However, it increased the risk of hospitalisation during the treatment period.
- Real-world improvements after bronchial thermoplasty resembled those in randomised trial arms. However, uncontrolled before–after changes do not reflect treatment effects.

**What are the implications of the main findings?**

- Bronchial thermoplasty is not currently considered an established alternative to biologic therapy; however, it may have a role in selected patients with severe, uncontrolled asthma.
- The improvements seen in uncontrolled studies should be interpreted with caution. This emphasises the need for standardised outcomes and long-term comparative research, including direct comparisons with biologic therapy.

**Abstract:**

**Introduction**: Bronchial thermoplasty (BT) reduces airway smooth muscle and may benefit severe asthma, but its role alongside biologics remains unclear. We assessed randomised and real-world evidence on efficacy and safety. **Methods**: We searched PubMed, Scopus, and Cochrane Library for RCTs and observational studies of BT in adults with severe uncontrolled asthma, analysing them separately. **Results**: Twenty studies were included (five RCTs, 15 observational). In RCTs, BT improved asthma-related quality of life (AQLQ;MD, 0.69; 95% CI 0.30–1.09), asthma control (ACQ; MD,−0.64; 95%CI, −1.10 to −0.19), and morning peak expiratory flow (MD, 20.62 L/min; 95% CI, 4.92–36.32), but did not improve rescue medication use or symptom-free-days. BT increased the risk of hospitalisation during the treatment period (RR, 3.50; 95%CI, 1.26–9.68), with no significant excess thereafter. Real-world studies showed improvements in AQLQ (MD,1.38; 95%CI, 0.97–1.80), ACQ (MD,−1.24; 95%CI, −1.86 to −0.62), and annualised exacerbation rates (MD, −3.79 exacerbations/patient-year; 95%CI, −6.98 to −0.61). Evidence suggested a possible, inconsistent corticosteroid-sparing effect; data on biologics and long-term safety were limited. **Conclusions**: BT improved asthma-related quality of life and control, mainly in trials without sham controls, but increased short-term hospitalisation risk. Real-world findings were similar but came from uncontrolled studies. BT is not an established alternative to biologics, but may suit carefully selected patients with severe asthma who cannot use or do not respond to them.

## 1. Introduction

Asthma is a prevalent chronic respiratory disease affecting children and adults, with an estimated global prevalence of 4–6%, corresponding to more than 250 million individuals worldwide [1]. Despite advances in pharmacological treatment, including optimisation of inhaled therapies, allergen avoidance, and management of comorbidities, asthma continues to contribute to morbidity and mortality, particularly among patients with severe disease. According to the Global Initiative for Asthma (GINA) and international respiratory societies, severe asthma is defined as asthma that remains uncontrolled despite optimised treatment with high-dose inhaled corticosteroids plus a second controller (a long-acting β-adrenergic agonist or a leukotriene receptor antagonist), or that requires frequent systemic corticosteroid use to maintain control [2,3].

Over the past decade, biologic therapies targeting key inflammatory pathways have revolutionised the management of severe asthma, reduced exacerbations and improved disease control and lung function. However, a subset of patients remains uncontrolled despite biologic therapy, while access to these therapies remains limited in many healthcare settings worldwide [2,4,5].

Bronchial thermoplasty (BT) is a non-pharmacological bronchoscopic procedure performed in three separate treatment sessions that delivers controlled radiofrequency energy to target the airway walls. The resulting reduction in airway smooth muscle mass is thought to reduce bronchoconstriction and airway hyperresponsiveness [2,6]. Histopathological studies have confirmed that this reduction persists for at least 2.5 years and may be associated with sustained clinical improvement [7]. Although randomised controlled trials (RCTs) have demonstrated improvements in quality of life and reductions in exacerbations, questions remain about the magnitude and consistency of the benefit, procedural safety, and the patient characteristics most likely to benefit from BT.

Several systematic reviews and meta-analyses have synthesised evidence from RCTs of BT. The Cochrane review by Torrego et al. (2014) [8] included three trials and reported modest improvements in quality of life, while highlighting the large placebo effect observed in the sham-controlled AIR2 trial [9]. More recently, Fong et al. (2023) [10] conducted a network meta-analysis comparing BT with biologics and found no significant differences in indirect comparisons across quality-of-life and exacerbation outcomes. Rayner et al. (2026), in the most comprehensive RCT-based meta-analysis to date, incorporating six trials and 573 patients as part of the AAAAI/ACAAI Joint Task Force guidelines, found that BT may improve asthma control, quality of life, and overall severe exacerbations, all with low certainty of evidence, while likely increasing procedural severe exacerbations and serious respiratory adverse events [11].

However, these meta-analyses have focused primarily on RCT evidence, which enrolled highly selected populations under controlled trial conditions. Over the past decade, a growing body of real-world observational evidence, including national registries, multicentre cohorts, and single-centre retrospective studies, has evaluated BT outcomes in broader and more heterogeneous populations, including patients concurrently treated with biologics, those with more severe airflow obstruction, and those from diverse geographic and healthcare settings. Yet these real-world data have not been quantitatively synthesised to provide an integrated assessment of outcomes following BT in routine clinical practice.

Therefore, this study had two objectives. First, we sought to independently synthesise the RCT evidence evaluating BT in adults with severe, uncontrolled asthma. Second, and as the primary novel contribution, we conducted the first quantitative synthesis of real-world observational evidence using single-arm pre–post meta-analyses of quality of life, asthma control, and exacerbation rates following BT. By integrating randomised and real-world evidence, we aimed to bridge the gap between controlled trial evidence and clinical practice and provide a comprehensive assessment of the potential role of BT in the current therapeutic landscape.

## 2. Materials and Methods

### 2.1. Eligibility Criteria

This systematic review and meta-analysis was conducted and reported in accordance with the Cochrane Handbook for Systematic Reviews of Interventions and the Preferred Reporting Items for Systematic Reviews and Meta-Analyses (PRISMA) 2020 statement [12]. Ethical approval was not required, as the review only analysed data from previously published studies. We registered the protocol with the International Prospective Register of Systematic Reviews (PROSPERO) under protocol CRD420251122282 (https://www.crd.york.ac.uk/PROSPERO/view/CRD420251122282, (accessed on 12 October 2026)).

We included studies of adults aged ≥ 18 years with severe asthma, defined by GINA or ERS/ATS criteria, who underwent bronchial thermoplasty (BT). Eligible designs comprised randomised controlled trials (RCTs) and longitudinal observational studies, including prospective and retrospective cohort studies and clinical or national registries. We included studies regardless of whether they had a comparator group; for uncontrolled observational studies, eligibility required extractable baseline and post-treatment data, or a directly reported change from baseline, for at least one prespecified outcome. We excluded case reports, editorials, narrative reviews, animal studies, and conference abstracts lacking extractable outcome data. We applied no restrictions on publication date, language, or follow-up duration.

When one cohort was a subset of another, the larger cohort was used in the primary analyses and the subset only in sensitivity analyses and narrative synthesis. When overlap was possible but partial and could not be quantified (shared centres between registries), both studies were retained, and the impact of overlap was assessed in sensitivity analyses.

### 2.2. Outcomes

The co-primary outcomes were changes in asthma-related quality of life, assessed by the Asthma Quality of Life Questionnaire (AQLQ), and asthma control, assessed by the Asthma Control Questionnaire (ACQ). For randomised controlled trials, secondary outcomes comprised morning peak expiratory flow (PEF), rescue medication use, symptom-free days, and hospitalisation. These were analysed separately for the peri-procedural treatment period (up to six weeks after the final BT session) and the post-treatment follow-up period. For the real-world synthesis, the prespecified pooled outcomes were changes in AQLQ, ACQ, and annual exacerbation rate from baseline. Other extracted outcomes, including biologic use, oral corticosteroid use, and adverse events and safety outcomes, were not pooled quantitatively because of insufficient consistency in outcome definitions, metrics or timing across observational studies, and were therefore summarised narratively. Studies using the mini-AQLQ, a validated short form sharing the AQLQ response scale and minimal important difference, were pooled with those using the full AQLQ; a sensitivity analysis excluded them.

### 2.3. Search Strategy and Study Selection

We conducted a comprehensive search across PubMed, Scopus, and the Cochrane Library from their inception until May 2025. The search employed two conceptual blocks: terms related to asthma and those related to bronchial thermoplasty. The detailed search strategies for each database are documented in the Supplementary Material (Table S1). References were imported into Rayyan (Rayyan Systems Inc., Cambridge, MA, USA), and two independent reviewers (MJL and MP) screened titles and abstracts in accordance with the eligibility criteria. Records deemed potentially eligible by at least one reviewer underwent full-text assessment by both authors independently. Any discrepancies identified at either stage were resolved through consensus, with the involvement of a third reviewer if necessary.

### 2.4. Data Extraction and Risk-of-Bias Assessment

Data were extracted using a predefined form and included the first author, year of publication, country, study design, sample size, patient demographics (age, sex, baseline FEV1% predicted, use of oral corticosteroids and biologics), BT procedural characteristics, follow-up duration, and outcome data. Two independent authors (MJL and MP) extracted the data; they resolved disagreements by consensus or, when consensus could not be reached, through adjudication by a third author.

We assessed bias using a tool matched to the study design. Randomised controlled trials were evaluated using the Cochrane Risk of Bias 2 (RoB 2) tool [13], which rates each trial as having a low risk of bias, some concerns, or a high risk of bias across five domains: bias arising from the randomisation process, deviations from intended interventions, missing outcome data, measurement of the outcome, and selection of the reported result. RoB 2 was applied separately to efficacy outcomes and to hospitalisation. We assessed observational studies without an internal comparator group, including uncontrolled before–after cohorts and registries, using the National Institutes of Health (NIH) Quality Assessment Tool for Before–After (Pre–Post) Studies with No Control Group [14,15]. As this tool does not prescribe a numerical scoring algorithm, an overall rating of Good, Fair, or Poor was assigned to each study based on the number of applicable criteria met (12 items, with item 12 not applicable to individual-level interventions such as BT): studies meeting 9 of 11 applicable criteria were rated Good, those meeting 7–8 were rated Fair, and those meeting 6 or fewer were rated Poor. Menzella et al. [16], the only observational study incorporating an internal comparator group, was instead assessed using the Newcastle–Ottawa Scale (NOS) for cohort studies [15], which rates studies across three domains—selection, comparability, and outcome assessment—for a maximum of 9 stars. Studies scoring 8–9 stars were rated High Quality, those scoring 7 were rated Moderate Quality, and those scoring 6 or fewer were rated Low Quality. Two independent authors (MJL and MP) performed all risk-of-bias assessments, with disagreements resolved by consensus or adjudication by a third author.

### 2.5. Statistical Analysis

For randomised controlled trials, dichotomous outcomes were summarised as risk ratios (RRs) and continuous outcomes as mean differences (MDs), with 95% confidence intervals (CIs), using DerSimonian–Laird random-effects models Review Manager (RevMan) version 5.4 (The Cochrane Collaboration, London, UK). AQLQ and ACQ analyses were stratified by sham versus non-sham control.

For observational studies, single-arm meta-analyses pooled mean changes from baseline using random-effects models with restricted maximum likelihood (REML) estimation of between-study variance, in R version 4.6.0 (R Foundation for Statistical Computing, Vienna, Austria) using the meta package (version 8.5.0).

When the standard deviation (SD) of the change was not reported, it was estimated from the baseline and follow-up SDs, assuming a pre–post correlation coefficient (r) of 0.5, with sensitivity analyses using 0.3 and 0.7, as suggested by the Cochrane Handbook. Medians with interquartile ranges (IQRs) were converted to means and SDs using the method of Wan et al. and Luo et al. [17,18]; when only the median and IQR width were reported, the median was used as the mean and the SD was estimated from the IQR width. Standard errors were converted to SDs (SD = SE × √*n*), and SDs were derived from 95% CIs using the t distribution. Exacerbation rates reported over other time windows were converted to rates per patient-year by multiplying means and SDs by the corresponding factor, assuming a constant rate. The proportion of studies requiring SD imputation or conversion is reported for each outcome in Supplementary Material (Tables S4–S12).

Statistical heterogeneity was assessed using Cochran’s Q test and the I^2^ statistic. A two-sided *p*-value < 0.05 was considered statistically significant.

Small-study effects were to be assessed by funnel plot inspection and Egger’s regression test for analyses including at least 10 studies. This applied only to the single-arm AQLQ analysis, for which results were interpreted cautiously given the high heterogeneity.

### 2.6. Sensitivity Analyses

Sensitivity analyses for the real-world outcomes used pre–post correlations of 0.3 and 0.7, the Hartung–Knapp–Sidik–Jonkman adjustment and leave-one-out analyses. To account for potential population overlap, AQLQ was re-analysed by excluding Torrego et al. [19], Burn et al. [20], or both Torrego et al. [19] and Menzella et al. [16], and Hatch et al. [21] replaced Langton et al. [22] for ACQ and exacerbations. Further analyses excluded mini-AQLQ studies (AQLQ) and studies with median/IQR-converted data (AQLQ, ACQ) (Figures S2–S4). Analyses not specified in the protocol were added during peer review.

## 3. Results

### 3.1. Study Characteristics

**Figure 1:**
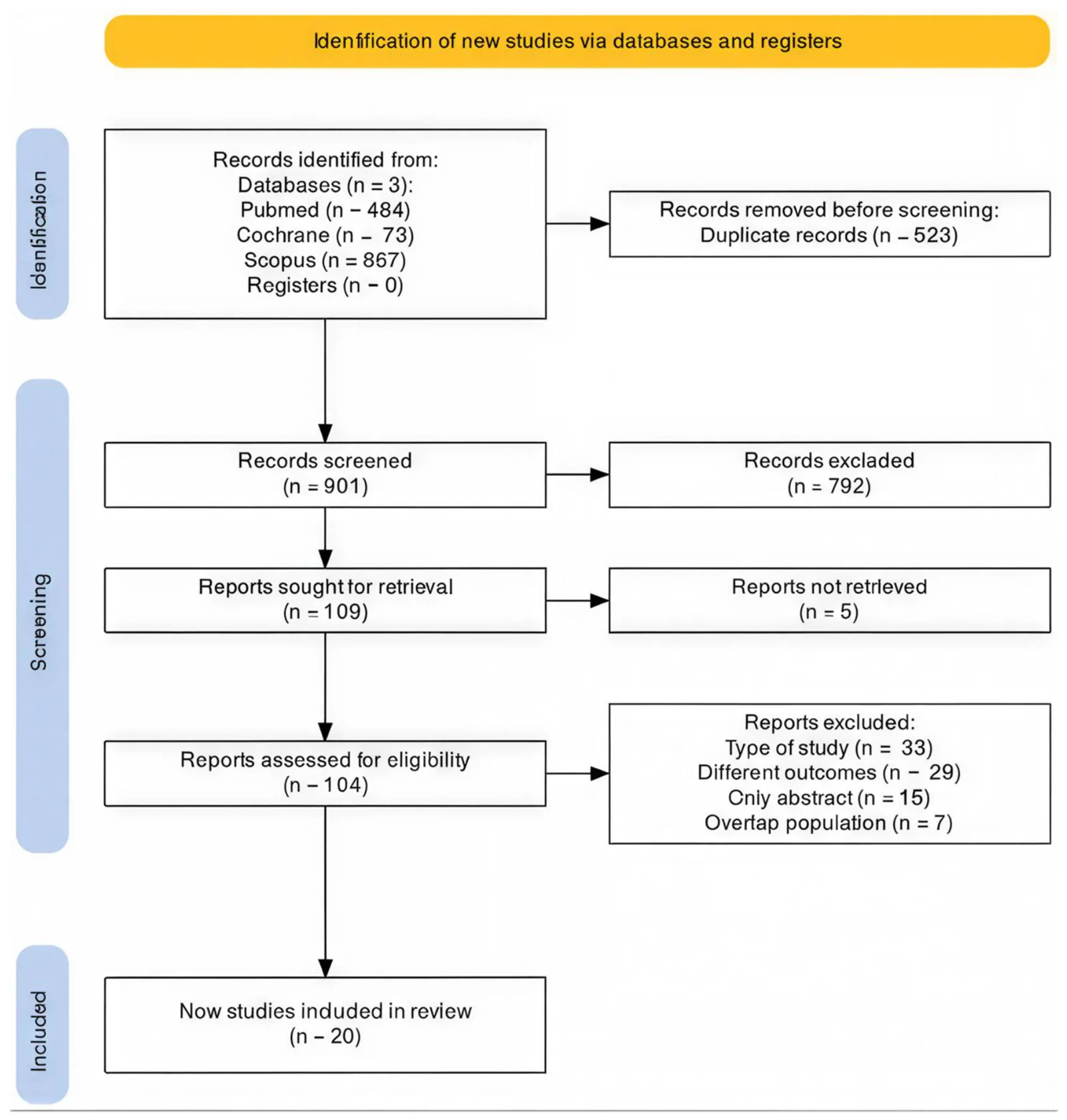
PRISMA flow diagram of study screening and selection.

The search strategy yielded 1424 articles or abstracts, as detailed in Figure 1.

After removing duplicate studies and screening for eligibility, 104 studies were assessed in full text. Of these, 20 met the inclusion criteria and were included in the qualitative review (five RCTs and 15 observational studies, including national and global registries as well as prospective and retrospective cohort series). Overall, 513 participants were randomised in the five RCTs (304 to BT and 209 to control), and 645 patients treated with BT were included in the 15 observational studies, some of which had partially overlapping populations. Hatch et al. [21] reports a subset of the Australian Bronchial Thermoplasty Register described by Langton et al. [22]; Menzella et al.’s [16] centre also contributes to the Bronchial Thermoplasty Global Registry (Torrego et al. [19]); and the latter also included UK and Australian centres that contributed to Burn et al. [20] and Langton et al. [22]. Patient-level linkage data were not available to quantify any overlap. To avoid double counting, Langton et al. [22] was used in the primary analyses and Hatch et al. [21] was used only in sensitivity analyses as a replacement; Hatch et al. [21] was retained for narrative outcomes, such as 5-year follow-up and biologic use. Menzella et al. [16] was retained because it provides a comparison with biologic therapies not available in the global registry. The impact of overlap was examined in sensitivity analyses excluding or replacing overlapping cohorts (Figures S2–S4). All included studies enrolled patients with severe, uncontrolled asthma consistent with GINA or ERS/ATS definitions [2,3]. Clinical outcomes were assessed using validated questionnaires, such as the AQLQ, ACQ, and objective measures, including lung function (FEV1) and exacerbation rates. Follow-up periods across all included studies ranged from 6 to 60 months. Studies were conducted primarily in Europe, Asia, and Australia, with further contributions from North and South America and global multicentre registries. Table 1 presents the study characteristics.

**Table 1:** Baseline characteristics of included studies (*n* = 20).

Study	Design	Country, Centres	BT N Analysed (Randomised) ^a^	Age, y (Mean ± SD)	Female	Baseline FEV1, % Pred	Baseline OCS	Baseline Biologics	Follow-Up, Mo
Castro 2010 (AIR2) [9]	RCT—sham	Multinational, 30 centres	190 ^a^ (196)	40.7 ± 11.89	57.40%	77.8 ± 15.65	3.70%	1.10%	12
Cox 2007 (AIR) [23]	RCT—standard care	Multinational (4 countries), 11 centres	49 ^a^ (56)	39.4 ± 11.2	56%	72.7 ± 10.4	NR	NR	12
Goorsenberg 2021 (TASMA) [24]	RCT—standard care (delayed treatment)	Netherlands/UK, 4 centres	18 ^a^ (20)	45 ± 14	85%	80.9 ± 20	20%	10%	6
Leroux 2024 (THERMASCORT) [25]	RCT—standard care, single-blind	France, 1 centre	13 ^a^ (15)	46.1 ± 10.4	87%	60.3 ± 16.3	NR	80% ^c^	15
Pavord 2007 (RISA) [26]	RCT—standard care, unblinded	Multinational (UK, Canada, Brazil), 8 centres	15	39.1 ± 13.0	60%	62.9 ± 12.2	53.30%	NR	12
Hatch 2023 [21]	Prospective registry cohort	Australia, 2 centres	51 ^a^ (53)	59.0 ± 11.8	51%	55.5 ± 18.8	53%	11.80%	60
Angsuwatcharakorn 2025 [27]	Retrospective cohort	Thailand, 1 centre	17 (NR)	54.9 ± 10.9	70.60%	76 (31) ^b^	47.10%	35.30%	12
Burn 2018 (UKSAR) [20]	Prospective multicentre registry	UK, 11 centres	86 ^a^ (131; 128 safety-evaluable)	43.7 ± 12.2 ^d^	69.8% ^d^	69.7 ± 21.7 (*n* = 82)	64.7% ^e^	16.70%	24
Soo (Chun Ian Soo) 2021 [28]	Retrospective cohort	Malaysia, 1 centre	17 ^a^ (20)	48 (33–55) ^b^	82%	59.2 ± 15.7	76%	18% ^c^	12
Frix 2019 [29]	Retrospective case series	Belgium, 1 centre	10	48 (38–58), mean	80%	57.3 ± 12.1 (pre-BD)	30%	0% (40% prior) ^c^	12
Hara 2023 [30]	Retrospective cohort	Japan, 1 centre	10 ^a^ (13)	NR(42–74)	60%	NR (subgroup only)	50%	NR	24
Inoue 2024 [31]	Retrospective cohort	Japan, 1 centre	15 ^a^ (19)	57 (20–74) ^b^	40%	56.5 (32.2–100) ^b^	40%	26.70%	6
Langton 2019 [22]	Registry-based cohort (Australian BT Register)	Australia, 4 centres	77 ^a^ (80)	57.7 ± 11.4	57.10%	55.8 ± 19.8	53.20%	0% (43% prior) ^c^	12
Madan 2021 [32]	Retrospective multicentre cohort	India, 4 centres	36	50.9 ± 11.5	69.40%	62.1 ± 18.5	22.20%	11.1% (+11.1% prior)	NR
Madsen 2019 [33]	Prospective cohort	Denmark, 1 centre	16	50 (31–57) ^b^	43%	62 (48–73) ^b^	NR	0%	24 ^g^
Menzella 2021 [16]	Retrospective cohort with biologic-arm comparator	Italy, 2 centres	30	57.6 ± 10.6	63%	71.2 ± 19.6	NR	27% ^c^	12
Nishi 2023 [34]	Retrospective cohort	Japan, 1 centre	21 ^a^ (23)	56.8 ± 12.3	85.70%	76.6 ± 24.8	47.60%	28.60%	12
Pretolani 2016 [35]	Prospective single-arm cohort	France, 1 centre	15	46.9 ± 11.9	53%	67.1 ± 19.5 (pre-BD)	67%	0% (67% prior) ^c^	3 (biopsy)/12 (clinical) ^f^
Seeley 2019 [36]	Prospective single-arm cohort	USA, 1 centre	25	49 ± 12.4	60%	72%	79% (any-dose, past year)/29% (chronic) ^h^	46% ^h^	12
Torrego 2021 (BTGR) [19]	Prospective multicentre registry	8 countries, 18 centres	157	49.8 ± 12.7	65.60%	NR	47.80%	9.60%	24

Abbreviations: AE, adverse event; AQLQ, Asthma Quality of Life Questionnaire; ACQ, Asthma Control Questionnaire; ACT, Asthma Control Test; ASM, airway smooth muscle; BT, bronchial thermoplasty; BTGR, Bronchial Thermoplasty Global Registry; FEV1, forced expiratory volume in 1 s; IQR, interquartile range; NR, not reported; OCS, oral corticosteroid; PEF, peak expiratory flow; RCT, randomised controlled trial; UKSAR, UK Severe Asthma Registry. Notes: a Number of patients in the study cohort (analysed for the primary outcome or with follow-up); the number randomised (RCTs) or treated with ≥1 BT session (observational studies) is shown in parentheses where reported and where it differs. The number contributing to each pooled analysis may be smaller and is given in Figure 2, Figure 3, Figure 4 and Figure 5 and the Supplementary Tables. Baseline characteristics refer to the population described in each source’s baseline table. b Reported in the source as the median (IQR or range), not the mean ± SD; c Percentage reflects biologic use prior to BT that had been stopped/discontinued before the procedure, not concurrent use during BT. Where the source gives both, both are shown; d Value applies to the registry’s broader eligible cohort (*n* = 126); e 64.7% (55/85) refers to baseline for the *n* = 86 efficacy cohort; f paired airway biopsies were taken pre-BT and at 3 months; clinical outcomes (ACT, AQLQ, exacerbations/hospitalisation/ICU/ED) were assessed at 3 and 12 months; g 16 patients were treated and assessed at 12 months, the timepoint used in the pooled analyses; 14 completed 24-month follow-up; h Percentages are as published; they are arithmetically consistent with *n* = 24 rather than the 25 patients enrolled.

### 3.2. Quality Assessment

The risk of bias in the five randomised controlled trials was independently assessed by two authors (MJL and MP) using the Cochrane Risk of Bias 2 (RoB 2) tool, separately for efficacy and hospitalisation outcomes. For efficacy outcomes, Castro et al. [9] was the only sham-controlled trial and was judged as being at low risk of bias, whereas the four trials [23,24,25,26] without a sham control raised some concerns, owing to the measurement of participant-reported outcomes in participants who were aware of their allocation, and, for Leroux et al. [25], missing outcome data. For hospitalisation, all trials were at low risk of bias except Leroux et al. [25] (some concerns, missing outcome data). No trial was judged as being at high risk in any domain (Figure S1, Supplementary Material).

We evaluated 14 studies using the NIH tool; Hatch et al. [21] was rated good. Six studies were rated fair [22,28,29,33,35,36] and seven were rated poor [19,20,27,30,31,32,34], most commonly because of limited or unverifiable follow-up, small sample sizes, or inconsistent outcome reporting. Inoue et al. [31] also had >20% attrition. All studies lacked blinded assessment and repeated measures; 11 [20,27,28,29,30,31,32,33,34,35,36] out of 14 were affected by small sample sizes. Menzella et al. [16], with a comparator, scored 5/9 on the Newcastle–Ottawa Scale, with no points awarded for the comparability domain due to the baseline imbalance between treatment groups. The item-level ratings are reported in the Supplementary Materials, in Tables S2 and S3.

### 3.3. Effects of Bronchial Thermoplasty in Randomised Trials

**Quality of life (AQLQ)**

**Figure 2:**
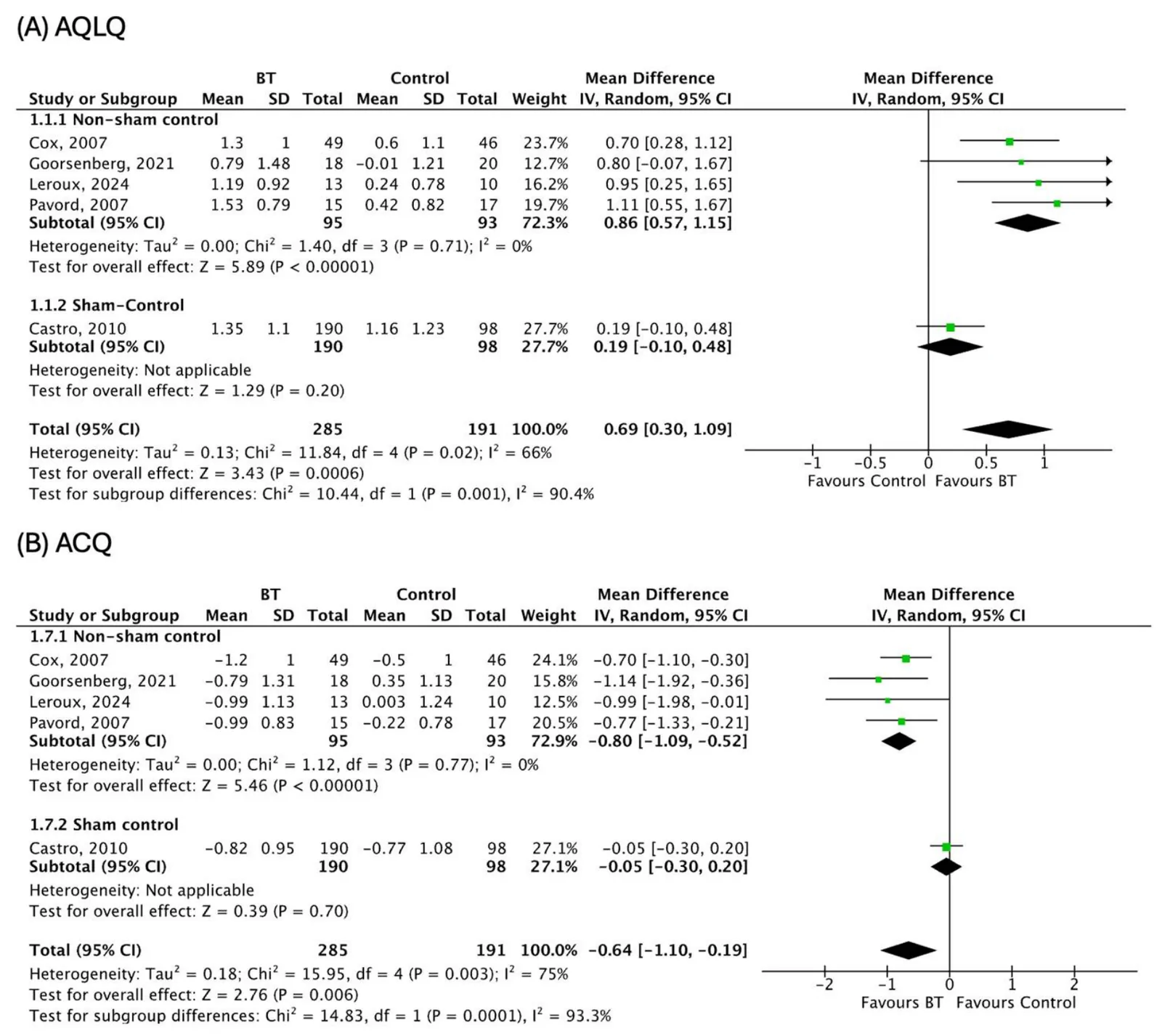
AQLQ (**A**) and ACQ (**B**) forest plot of randomised trials [9,23,24,25,26].

Five RCTs [9,23,24,25,26] evaluated asthma quality of life using the AQLQ, involving 476 patients (*n* = 285 in the BT group and *n* = 191 in the control group). Compared with the control, BT was associated with a significant improvement in AQLQ score (MD = 0.69, 95% CI [0.3, 1.09], *p* = 0.0006; I^2^ = 66%) (Figure 2A).

**Asthma control questionnaire (ACQ)**

All RCTs [9,23,24,25,26] included 476 patients (*n* = 285 in the BT group and *n* = 191 in the control group) and assessed asthma control using the ACQ. Overall, BT was associated with a significant improvement in ACQ scores compared with the control (MD = −0.64, 95% CI [−1.10, −0.19], *p* = 0.006; I^2^ = 75%) (Figure 2B).

**Morning peak expiratory flow (PEF)**

**Figure 3:**
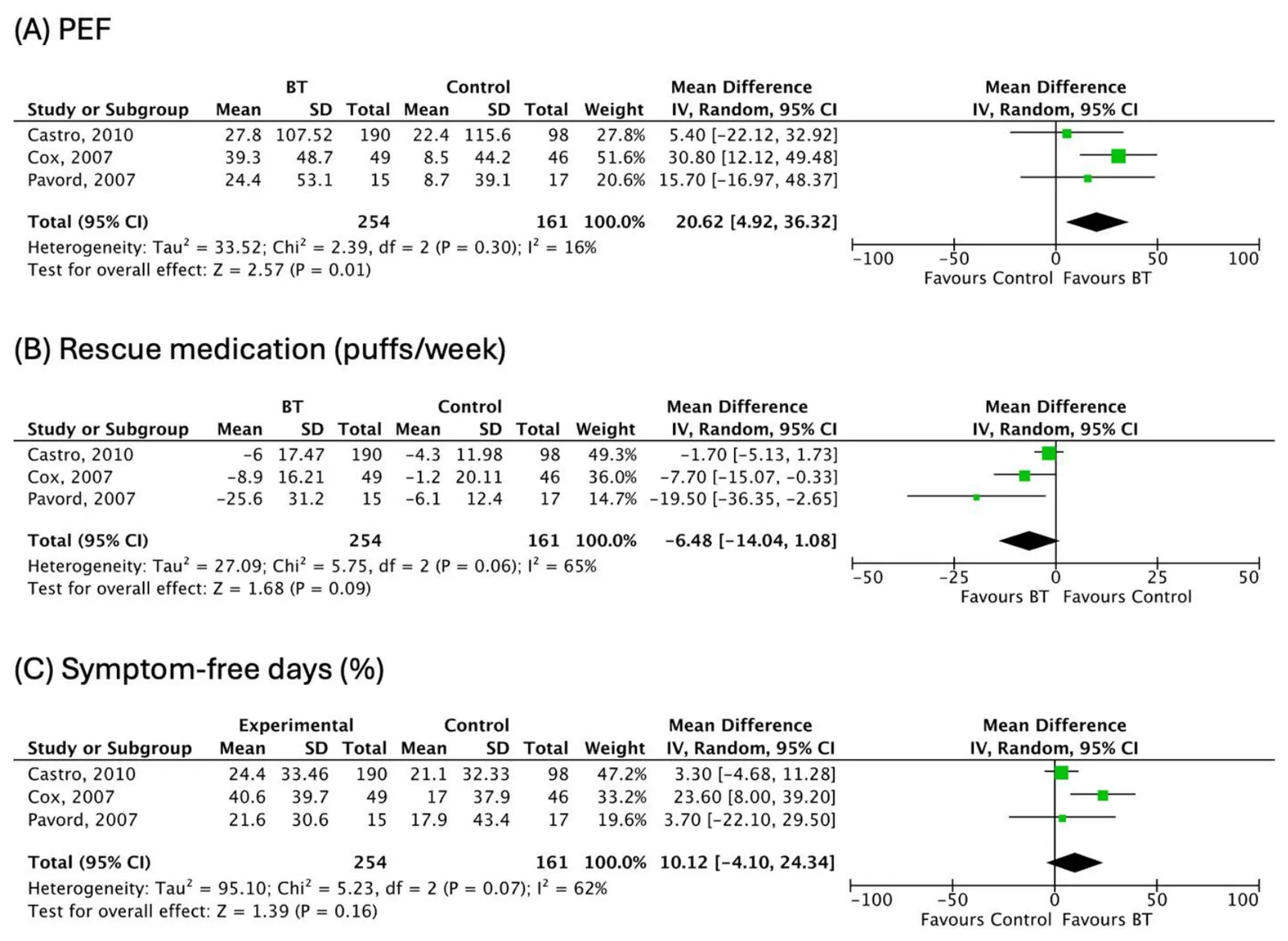
PEF (**A**), rescue medication (**B**) and symptom-free days (**C**) forest plot of randomised studies [9,23,26].

Three RCTs [9,23,26] involving 415 patients (*n* = 254 in the BT group and *n* = 161 in the control group) assessed peak expiratory flow (PEF). BT was associated with a statistically significant improvement in PEF compared with the control (MD = 20.62 L/min, 95% CI [4.92, 36.32], *p* = 0.01; I^2^ = 16%) (Figure 3A).

**Rescue medication (puffs/week)**

Three RCTs [9,23,26] involving 415 patients (*n* = 254 in the BT group and *n* = 161 in the control group) assessed changes from baseline in rescue medication use. BT was associated with a non-significant reduction compared with the control (MD = −6.48 puffs/week, 95% CI [−14.04, 1.08], *p* = 0.09; I^2^ = 65%) (Figure 3B).

**Symptom-free days (%)**

In three RCTs [9,23,26] including 415 participants (*n* = 254 in the BT group and *n* = 161 in the control group), there was a non-significant increase in symptom-free days in the BT group versus the control, with moderate heterogeneity (MD = 10.12, 95% CI [−4.10, 24.34]; *p* = 0.16; I^2^ = 62%) (Figure 3C).

**Hospitalisation due to respiratory events**

**Figure 4:**
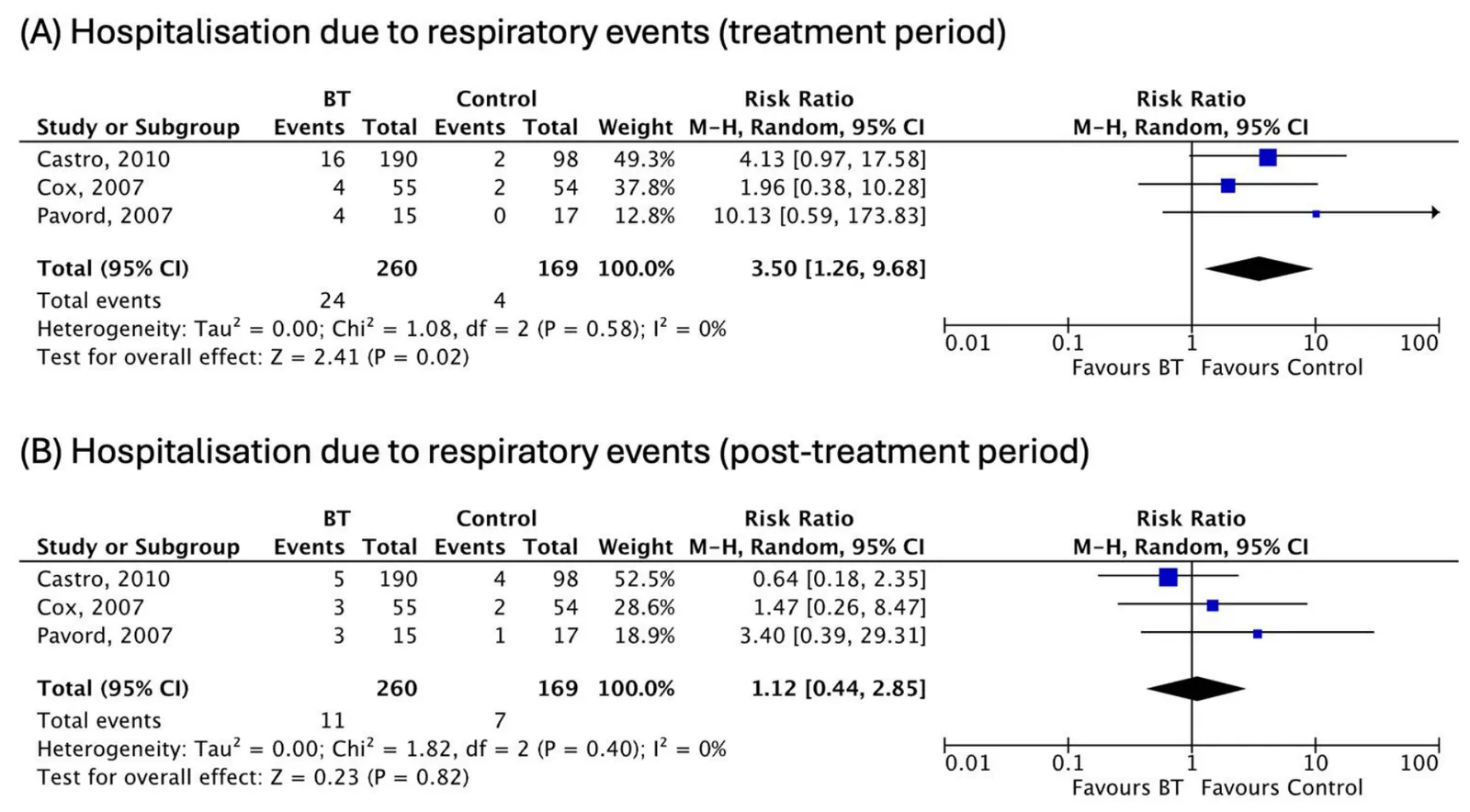
Forest plots of hospitalisation due to respiratory events in randomised trials: (**A**) treatment period; (**B**) post-treatment period [9,23,26].

Three RCTs [9,23,26] reported hospitalisations for respiratory events, distinguishing between the treatment period (up to 6 weeks after the procedure) and the post-treatment period. During the treatment period, 24 of 260 patients in the BT group (9.2%) required hospitalisation, compared with 4 of 169 in the control group (2.3%), corresponding to a significantly increased risk in the BT group (RR 3.50, 95% CI 1.26 to 9.68; *p* = 0.02; I^2^ = 0%). In the post-treatment period, hospitalisation rates were similar (4.2% in the BT group vs. 4.1% in controls; RR 1.12, 95% CI 0.44 to 2.85; *p* = 0.82; I^2^ = 0%) (Figure 4).

### 3.4. Real-World Observational Evidence

**Figure 5:**
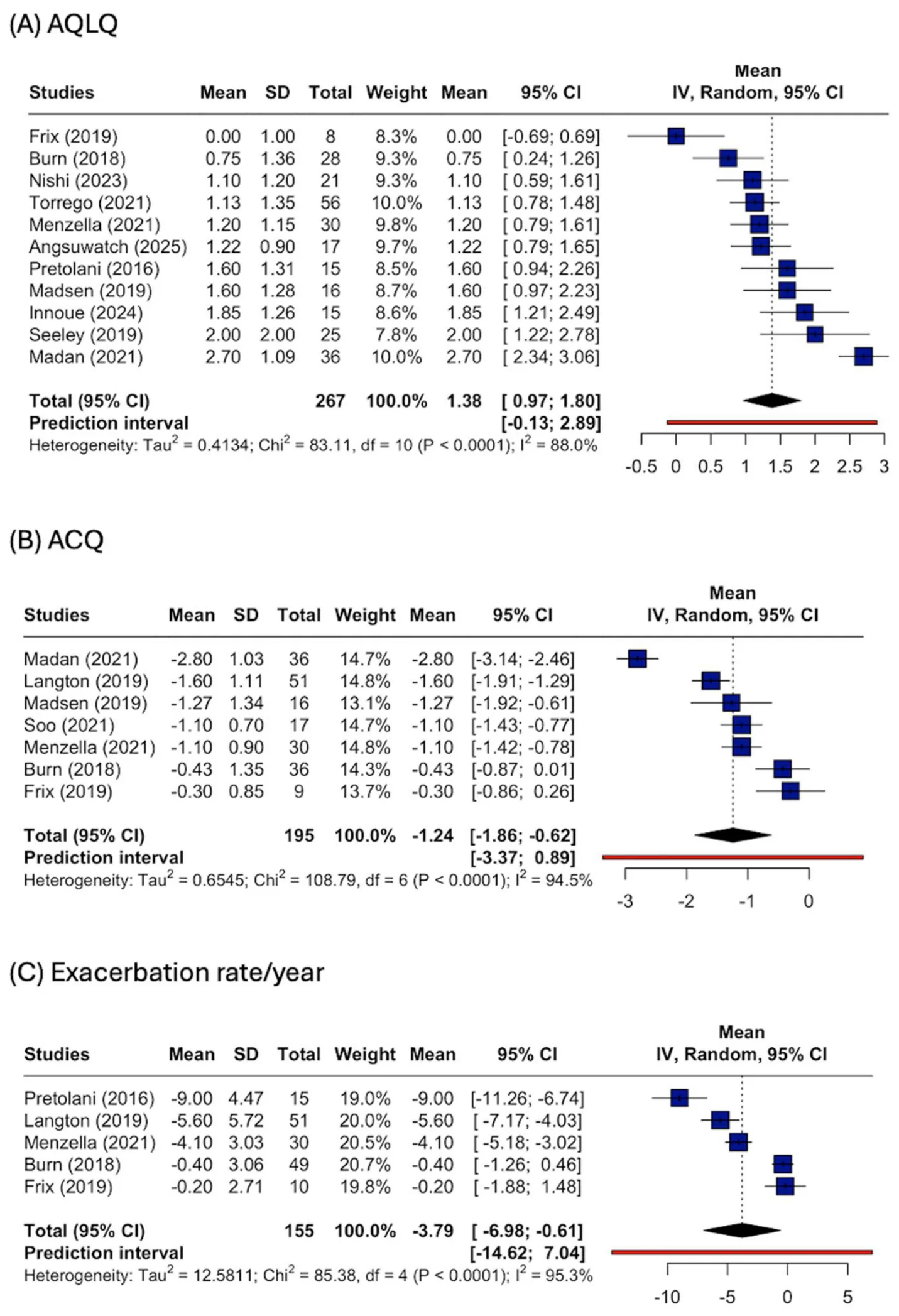
AQLQ (**A**) [16,19,20,27,29,31,32,33,34,35,36], ACQ (**B**) [16,20,22,28,29,32,33], and exacerbation rate/year (**C**) [16,20,22,29,35] of observational studies.

Quality of life (AQLQ): Eleven observational studies [16,19,20,27,29,31,32,33,34,35,36], including 267 patients, evaluated changes in AQLQ following bronchial thermoplasty. A pooled analysis of within-study changes showed a mean improvement in AQLQ score of 1.38 points (95% CI 0.97–1.80), although substantial between-study heterogeneity was observed (I^2^ = 88%) (Figure 5A). Results were materially unchanged when alternative pre–post-correlation coefficients were assumed (r = 0.3 and 0.7), after HKSJ adjustment, and in overlap, leave-one-out and other sensitivity analyses (Figure S2).

Asthma Control Questionnaire (ACQ): Seven observational studies [16,20,22,28,29,32,33] involving 195 patients assessed changes in ACQ scores after bronchial thermoplasty. Patients showed a pooled mean reduction of 1.24 points from baseline (95% CI −1.86 to −0.62), with considerable heterogeneity across studies (I^2^ = 94.5%) (Figure 5B). Similar results were observed with different assumed pre–post correlation coefficients (r = 0.3 and 0.7), after HKSJ adjustment, and in overlap and leave-one-out analyses (Figure S3).

Annual exacerbation rate: Five observational studies [16,20,22,29,35] involving 155 patients assessed changes in the annual exacerbation rate after bronchial thermoplasty. A pooled analysis of within-study changes showed a mean reduction of 3.79 exacerbations per patient-year (95% CI −6.98 to −0.61). Between-study heterogeneity was considerable (I^2^ = 95.3%), with a prediction interval ranging from −14.62 to 7.04 exacerbations per patient-year (Figure 5C). Results were similar with r = 0.3 and 0.7, but the estimate was no longer significant after HKSJ adjustment or after omitting any one of three studies [16,22,35] (Figure S4).

**Biologic use**

Two studies reported omalizumab discontinuation after BT. Seeley et al. [36] found that omalizumab use fell from 46% to 17% of the cohort (*n* = 25) at 1 year (*p* < 0.05). Angsuwatcharakorn et al. [27] reported that six of 17 patients (35.3%) were on omalizumab before BT; all six discontinued treatment within 12 months.

In the 5-year cohort of Hatch et al. [21] (*n* = 51), 21 patients started an IL-5–targeted antibody > 12 months after BT, and 30 did not. ACQ improvement did not differ between subgroups (baseline–60 months: 1.5 ± 1.0 vs. 1.2 ± 1.2, *p* = 0.41), nor did exacerbation frequency, although the difference in exacerbation frequency approached significance (1.5 ± 3.3 vs. 3.8 ± 4.2, *p* = 0.052). The authors attributed the difference in exacerbation frequency to higher baseline exacerbation rates in the non-biologic group rather than a treatment effect.

Menzella et al. [16] allocated 199 patients to benralizumab (*n* = 32), mepolizumab (*n* = 83), omalizumab (*n* = 54) or BT (*n* = 30) based on drug-specific eligibility rather than randomisation, with BT reserved for patients ineligible for, unresponsive to, or unwilling to receive biologics. FEV1 improved in all biologic arms but not after BT (+5.7%, *p* = 0.29), and the proportion weaned off OCS was lowest with BT (53% vs. 69–89%; *p* = 0.013), although the relative reduction in mean OCS dose after BT (−76%, *p* < 0.0001) was second only to that with mepolizumab (−90.2%). However, reductions in hospitalisations (−93.7%, *p* = 0.001) and exacerbations (−73.5%, *p* < 0.0001) after BT were comparable to those with mepolizumab and omalizumab. These between-treatment comparisons were descriptive and should be interpreted cautiously given non-randomised treatment allocation, baseline differences between treatment groups, and the potential for confounding by indication.

**Oral corticosteroid use**

Thirteen of the 15 real-world studies reported baseline maintenance OCS use as a proportion of the cohort, with figures ranging from 22.2% (8/36) in Madan et al. [32] to 76% (13/17) in Soo et al. [28]. Madsen et al. [33] excluded patients on maintenance OCS > 10 mg/day and reported only rescue OCS courses. Menzella et al. [16] reported only the proportion of the whole cohort on maintenance OCS (49%); in the BT arm, the mean dose fell from 14.7 ± 10.2 to 3.5 ± 4.9 mg/day (*p* < 0.0001).

Several studies reported a significant decline in the proportion of the whole cohort on OCS after BT: Torrego et al. [19] reported a decline from 47.8% to 24.8% at 2 years (*p* = 0.0002), and Soo et al. [28] reported 59% (10/17) being OCS-free at 12 months. Burn et al. [20] and Seeley et al. [36] found no significant change (64.7% to 60%, *p* = 0.56; 29% to 21%, *p* = 0.43). Among patients remaining on OCS, dose fell significantly in some studies (Angsuwatcharakorn et al. [27] 5 to 1.25 mg/day, *p* = 0.03; Pretolani et al. [35] 31.5 to 13.8 mg/day, *p* = 0.002) but not in the two largest cohorts (Torrego et al. [19] 21.0 to 15.2 mg/day, *p* = 0.15; Burn et al. [20]: 25 to 20 mg/day, *p* = 0.44).

Complete weaning was reported by Langton et al. [22] in 48.8% (20/41) of baseline OCS users by 6 months, with a lower baseline dose predicting successful weaning (56% if ≤10 mg/day vs. 38% if >10 mg/day, among subgroups defined by baseline dose); Hatch et al. [21] reported 41% weaned by 6 months, and Angsuwatcharakorn et al. [27] reported that 50% (4/8) of baseline OCS users stopped entirely.

In contrast, Madsen et al. [33] reported a significant increase in rescue (not maintenance) OCS courses after BT, from 0% at baseline to 35.7–42.9% across follow-up (*p* ≤ 0.009). Frix et al. [29], Hara et al. [30], Inoue et al. [31], and Nishi et al. [34] reported smaller or less complete OCS datasets (cohorts of 10–21 patients, mostly non-significant or non-quantified changes), precluding reliable conclusions regarding the effect of BT on OCS use in these cohorts.

**Adverse events (AE) and Safety**

Safety reporting was markedly inconsistent across the 15 real-world studies, limiting quantitative synthesis and interpretation of the incidence of uncommon adverse outcomes. Deaths were explicitly reported as zero in three studies (Soo et al. [28] Inoue et al. [31], and Torrego et al. [19]). Angsuwatcharakorn et al. [27] reported no events under a composite “severe complications” definition (death or mechanical-ventilation-requiring respiratory failure), which does not allow the absence of death to be established independently. Two further studies incidentally reported a single death each as a reason for loss to follow-up rather than as a safety finding (a motor vehicle accident (Hatch et al. [21]) and a death of unstated cause within two years of treatment (Hara et al. [30])); the remaining nine studies did not address deaths at all. Peri-procedural respiratory AEs, where reported, were common and broadly consistent between the two largest registries (44.5% in Burn et al. [20] and 45.2% in Torrego et al. [19]) and mostly involved wheeze, cough, dyspnoea, and infection, with events concentrated during the treatment period. Only Torrego et al. [19] used a formal severe AE definition (28.0% during treatment, falling to 0–0.8% by 1–2 years of treatment); three studies (Langton et al. [22], Nishi et al. [34], Pretolani et al. [35]) reported no safety data. Hatch et al. [21] reported mild, localised radiological bronchiectasis on HRCT 5 years after BT in 23 of 47 patients (49%); in 34 patients with a baseline scan, the modified Reiff score increased by 1.0 ± 1.4 (*p* < 0.001), although no patient had clinical features of bronchiectasis. Overall, the available observational evidence indicates that respiratory adverse events were concentrated around the treatment period; however, variation in AE definitions, ascertainment methods, and reporting of adverse events substantially limit reliable estimates of longer-term safety.

## 4. Discussion

This meta-analysis synthesised randomised and real-world evidence on bronchial thermoplasty (BT) in severe, uncontrolled asthma in the biologic therapy era. Randomised trials showed improvements in quality of life, asthma control and morning peak expiratory flow, mainly in trials without a sham control, while rescue medication use, and symptom-free days did not improve significantly. Although the pooled AQLQ and ACQ differences exceeded the minimal clinically important difference of 0.5 points, their confidence intervals did not exclude smaller effects. In AIR2 [9], the only sham-controlled trial, the between-group AQLQ difference was 0.19 points. These findings suggest that a clinically important benefit is therefore plausible, but its magnitude remains uncertain and may have been overestimated in the unblinded trials. Importantly, BT was associated with an increased risk of hospitalisation during the treatment period, but not thereafter. In observational studies, the changes in quality of life and asthma control after BT were similar to those observed in the BT arms of randomised trials, although with substantial heterogeneity and important methodological limitations. Overall, the evidence suggests that BT may provide benefit in selected outcomes for carefully selected patients, but these benefits must be weighed against the short-term procedural risk and the uncertainty inherent in much of the real-world evidence.

The difference between our ACQ and AQLQ estimates and those reported by Rayner et al. [11] warrants consideration. Rayner et al. [11], based on the same trials, reported modest but significant improvements (MD = −0.37 and 0.55, respectively), which likely reflects methodological differences, including their use of adjusted ANCOVA/REML analysis compared with our unadjusted change-from-baseline analysis using a DerSimonian–Laird random-effects model in RevMan. Rayner et al. [11] also included one additional Chinese-language trial (Lv 2021 [37]) in some outcomes. Because this trial was not identified in our database searches and its full text could not be retrieved, we could not independently assess its eligibility or extract its data to be included in our synthesis. Given its small weight in the published analysis, its omission is unlikely to materially alter the overall interpretation of the RCT evidence. Using unpublished trial data, Rayner et al. [11] also reported a modest reduction in severe exacerbations (IRR 0.81, 95% CI 0.68–0.97). The improvement in PEF should also be interpreted in context, given its effort dependence and relatively limited clinical relevance compared with patient-centred outcomes such as exacerbations, quality of life, and hospitalisation.

Real-world observational estimates were derived predominantly from uncontrolled before-and-after comparisons and are therefore not directly comparable to the between-group differences reported in randomised trials. Changes within groups may also reflect placebo and procedural effects, regression to the mean, selection bias, disease fluctuation and changes in background therapy, including initiation or switching of biologics during follow-up in some cohorts [21,31,34]. Notably, the sham arm of AIR2 also improved substantially [9]. When contrasted with the corresponding within-group changes in the BT arms of randomised trials, the improvements seen in routine practice were of similar magnitude. Consequently, the real-world synthesis should be viewed as illustrating how patients change after BT in clinical settings, rather than as proof of its efficacy. High heterogeneity across studies further limits confidence in these estimates.

Nonetheless, real-world observational findings offer valuable insights into routine practice, including longer follow-up in some cohorts and treatment patterns, such as changes in oral corticosteroid and biologic use, which RCTs capture less easily. Although controlled follow-up did not extend beyond 12 months, the longest observational cohort reported sustained improvement at 5 years without a corresponding change in spirometry. However, this cohort was uncontrolled, and 21 of 51 patients started a biologic during follow-up [21]. Thus, it remains unknown whether or not BT modifies the long-term course of severe asthma. These findings should be considered hypothesis-generating and complementary to, rather than a substitute for, comparative randomised evidence.

The observational data on corticosteroids and biologic therapy also provide insight into the potential role of BT within contemporary severe asthma management. Several cohorts reported reductions in maintenance OCS use, dose, or complete discontinuation following BT, but these findings were inconsistent, with one study reporting increased rescue OCS courses. The largest prospective real-world cohort (PAS2; *n* = 284), which could not be included because its full text was not available, reported a reduction in the proportion of patients on maintenance OCS from 19.4% to 9.7% at 5 years [38]. Differences in baseline OCS exposure, definitions, follow-up, and reporting completeness preclude firm conclusions regarding a generalisable OCS-sparing effect. Similarly, biologic treatment patterns suggest that BT may have a complementary or sequential role alongside biologic therapy, with some patients discontinuing biologics after BT and others subsequently initiating biologic treatment. Menzella et al. [16] provided the only direct comparison with biologic therapies, but BT was preferentially used in patients who were ineligible for, unresponsive to, or unwilling to receive biologics. Accordingly, these findings cannot establish comparative effectiveness because of baseline imbalance and likely confounding by indication. In the absence of head-to-head randomised trials, the relative effectiveness of BT and biologic therapy remains unknown.

In the RCTs, BT increased the risk of hospitalisation during treatment and respiratory adverse events were more frequent than in controls, typically shortly after bronchoscopy. Safety reporting in real-world studies was substantially less consistent than efficacy reporting. Only three studies explicitly documented zero deaths, while nine did not address mortality, thereby constraining the ability of real-world data to validate the peri-procedural safety signal observed in the trials. Taken together with the RCT findings, the available evidence suggests that respiratory adverse events are concentrated around treatment, but inconsistent definitions and ascertainment prevent reliable estimation of longer-term safety. Long-term radiological safety is uncertain. In the BT10+ study [39], which re-evaluated AIR [23], AIR2 [9], and RISA [26] participants a median of 12 years after treatment, 13% of BT-treated AIR2 [9] participants had bronchiectasis on HRCT, and 7% of those without baseline bronchiectasis developed it, with most cases being mild. Hatch et al. [21] found mild localised radiological bronchiectasis in nearly half of patients at 5 years, without clinical features. The significance of these findings remains unknown. No included study evaluated repeat BT, and a reduction in airway smooth muscle should not be equated with a reversal of airway remodelling, whose long-term consequences remain unclear.

### 4.1. Strengths

This review combines RCT and real-world evidence while maintaining a clear distinction between comparative efficacy and uncontrolled treatment-response data. We used design-appropriate risk-of-bias assessment tools (RoB 2, the NIH pre–post tool, the Newcastle–Ottawa Scale), performed sensitivity analyses using different pre–post correlation assumptions, Hartung–Knapp–Sidik–Jonkman adjustment and leave-one-out analyses, and explicitly examined potential cohort overlap. Our safety assessment also distinguished studies that explicitly reported zero events from those that did not report the outcome, avoiding the assumption that non-reporting equated to the absence of events. 

### 4.2. Limitations

The principal limitation in this real-world evidence is the substantial reliance on uncontrolled and single-arm observational data for several clinically important outcomes. Of the 14 real-world studies assessed with the NIH pre–post tool, only one (Hatch et al. [21]) was rated good, six were rated fair, and seven were rated poor, most often reflecting limited or unverifiable follow-up, small sample sizes, or inconsistent outcome reporting. Menzella et al. [16], the sole comparator study, scored five of nine on the Newcastle–Ottawa Scale, with no stars awarded for comparability given the unadjusted, non-randomised comparison across four treatment arms with significant baseline imbalance, reinforcing the concern regarding confounding by indication. Predictors of response remain poorly established. Greater baseline severity was associated with greater improvement [22], but this is also consistent with regression to the mean, and other candidate predictors were inconsistent or derived from small, exploratory analyses. Variability and inconsistent variable definitions prevented combining biologic-use and safety data. Several cohorts may have overlapped (Hatch et al. [21] with Langton et al. [22], and the global registry of Torrego et al. [19] with Burn et al. [20], Langton et al. [22] and Menzella et al. [16]), and patient-level linkage data were unavailable to quantify this; however, excluding or replacing overlapping cohorts did not materially change the AQLQ or ACQ estimates. We did not perform a formal GRADE assessment: the RCT evidence has its own GRADE rating [11], and single-arm real-world data are unsuitable for comparative certainty ratings. Instead, interpretation of the observational evidence was guided by study-level risk of bias, substantial heterogeneity, and wide prediction intervals.

### 4.3. Implications for Clinical Practice

These findings support a selective and individualised role for BT in severe, uncontrolled asthma, rather than routine use across all patients eligible for advanced therapy. BT may be considered in patients who remain uncontrolled despite optimised treatment, particularly when biologic therapy is unavailable, unsuitable, or ineffective. Clinical decisions should balance potential improvements in patient-centred outcomes against the short-term procedural risk and uncertainty surrounding long-term safety and comparative effectiveness.

### 4.4. Future Research Directions

Future research should focus on standardised safety reporting, prospective direct comparisons of BT with biologic therapies, and long-term evaluation of OCS and biologic-sparing effects. Studies should also investigate treatment sequencing and predictors of response to identify patients most likely to derive clinically meaningful benefit from BT.

## 5. Conclusions

In randomised trials, bronchial thermoplasty was associated with improvements in quality of life, asthma control and morning peak expiratory flow, but not in rescue medication use, or symptom-free days. The observed improvements in quality of life and asthma control were derived mainly from trials without a sham control, and their true magnitude therefore remains uncertain. It is associated with a temporary increase in respiratory hospitalisations during the treatment period, without evidence of persistent excess after treatment. Real-world studies reported improvements in quality of life, asthma control and exacerbation rates after BT, but these uncontrolled before-and-after changes cannot be interpreted as treatment effects and are limited by substantial heterogeneity. Observational evidence also suggests a possible but inconsistent OCS-sparing effect, while the role of BT relative to biologic therapy remains uncertain. Overall, current evidence supports neither routine BT use across the severe asthma population nor its establishment as an alternative to biologic therapy. BT may have a role in carefully selected patients with severe uncontrolled asthma, particularly when biologic therapy is unsuitable or has not achieved adequate disease control, although the benefits of BT in these specific circumstances have not been established by robust comparative evidence. Treatment decisions should be individualised, balancing potential patient-centred benefits against procedural risk and uncertainty regarding long-term safety and comparative effectiveness. Future research should prioritise standardised safety reporting, direct comparisons with biologic therapies, long-term treatment-sparing outcomes, and identification of patients most likely to benefit from BT.

## Data Availability

The authors confirm that the data supporting the findings of this study are available in the article and its Supplementary Materials. Further enquiries can be directed to the corresponding author.

## Supplementary Materials

The following supporting information can be downloaded at https://www.mdpi.com/article/10.3390/arm94050077/s1, Table S1: Search queries used in the different electronic bibliographic databases; Figure S1: Risk of bias (RoB 2) in the randomised controlled trials; Table S2: Methodological quality assessment of single-arm studies (NIH Before–After Tool); Table S3: Newcastle–Ottawa Scale for comparative cohorts; Figure S2: Sensitivity analyses for AQLQ in real-world studies; Figure S3: Sensitivity analyses for ACQ in real-world studies; Figure S4: Sensitivity analyses for annual exacerbation rate in real-world studies; Tables S4–S9: Data extraction for the RCT meta-analyses (ACQ, AQLQ, morning PEF, rescue medication, symptom-free days and hospitalisation); Tables S10–S12: Data extraction for the single-arm meta-analyses of real-world studies (AQLQ, ACQ and exacerbation rate).
