# Supplementary material for: Bronchial Thermoplasty in Severe Asthma: Bridging Randomised and Real-World Evidence: A Systematic Review and Meta-Analysis
Source: Adv Respir Med. 2026 Oct 21;94(5):77. doi: 10.3390/arm94050077 (PMC13701635; doi:10.3390/arm94050077)
Supplement: Supplementary file 1 [file arm-94-00077-s001.zip › arm-4572302-supplementary.pdf]

SUPPLEMENTARY MATERIAL

Table S1: Search queries used in the different electronic bibliographic databases

Database	**Search query**	**Time of search**
PUBMED	(“Asthma” OR “Bronchial asthma”)<br><br>(Thermoplasty OR “Bronchial Thermoplasty” OR “Radiofrequency ablation”)	May, 30, 2025
SCOPUS	(TITLE-ABS-KEY(“Asthma” OR “Bronchial asthma”))<br><br>(TITLE-ABS-KEY(Thermoplasty OR “Bronchial Thermoplasty” OR “Radiofrequency ablation”))	May 30, 2025
COCHRANE	(“Asthma” OR “ Bronchial asthma”)<br><br>(Thermoplasty OR “Bronchial Thermoplasty” OR “Radiofrequency ablation”)	May 30, 2025

Figure S1: Risk of bias (RoB 2) in the randomised controlled trials. (A) Efficacy outcomes (AQLQ, ACQ, PEF, rescue medication, symptom-free days). D4 was rated 'some concerns' in trials without a sham control, as outcomes were participant-reported; (B) Hospitalisation. D4 was rated low risk in all trials, as hospitalisation is an objective outcome. In both panels, D3 was rated 'some concerns' for Leroux 2024 because some randomised participants were not analysed.

A) B)

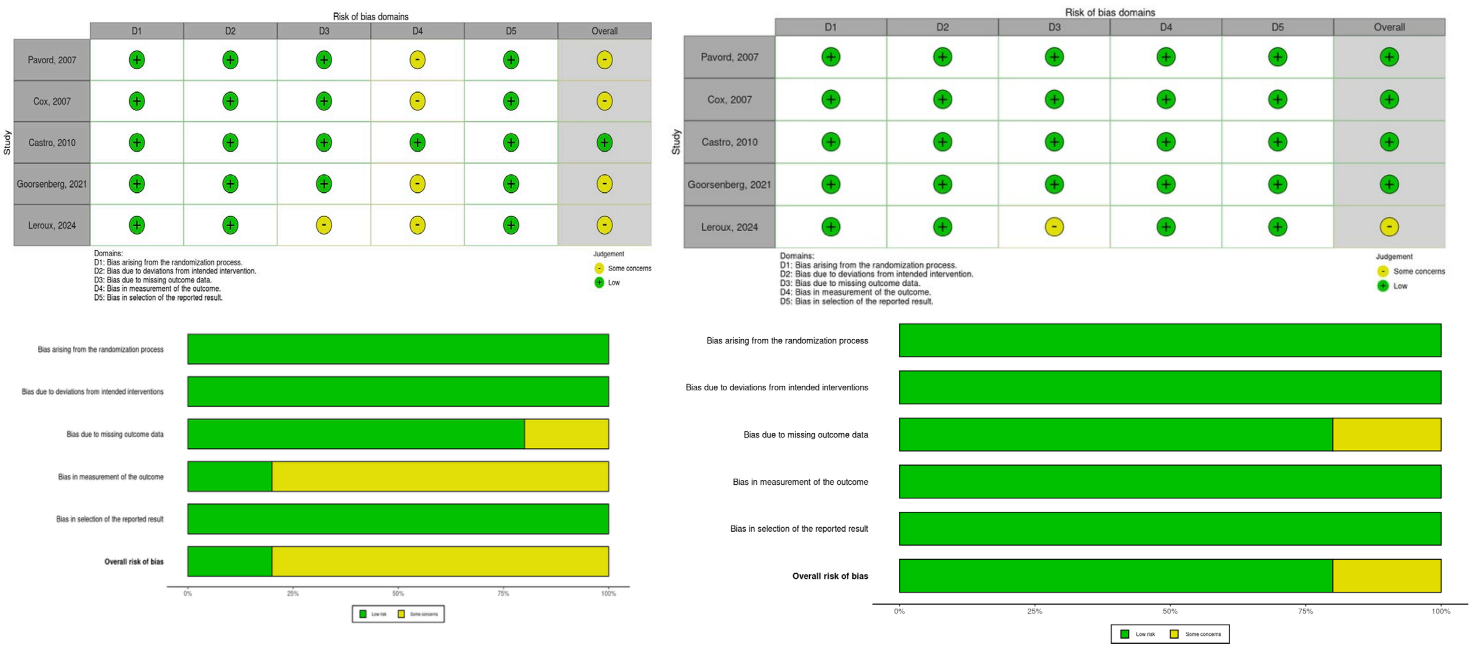

Table S2: Methodological Quality Assessment of Single-Arm Studies (NIH Before-After Tool)

Y = Yes; N = No; CD = Cannot Determine; NA = Not Applicable

Study (Author, Year)	Q1	Q2	Q3	Q4	Q5	Q6	Q7	Q8	Q9	Q10	Q11	Q12	Overall Quality Rating
**Pretolani (2016)**	Y	Y	CD	CD	N	Y	Y	N	Y	Y	N	NA	Poor
**Burn (2018)**	Y	Y	Y	CD	N	Y	N	N	N	Y	N	NA	Poor
**Frix (2019)**	Y	CD	N	CD	N	Y	Y	N	Y	Y	N	NA	Poor
**Langton (2019)**	Y	Y	Y	Y	CD	Y	Y	N	Y	Y	N	NA	Fair
**Madsen (2019)**	Y	Y	Y	CD	N	Y	Y	N	Y	Y	N	NA	Fair
**Seeley (2019)**	Y	Y	CD	CD	N	Y	Y	N	Y	Y	N	NA	Poor
**Chun Ian Soo (2021)**	Y	Y	Y	CD	N	Y	Y	N	Y	Y	N	NA	Fair
**Madan (2021)**	Y	Y	Y	CD	N	Y	Y	N	CD	Y	N	NA	Poor
**Torrego (2021)**	Y	CD	Y	CD	N	N	N	N	N	Y	N	NA	Poor
**Hara (2023)**	Y	Y	Y	N	N	Y	Y	N	N	Y	N	NA	Poor
**Hatch (2023)**	Y	Y	Y	Y	Y	Y	Y	N	Y	Y	N	NA	Good
**Nishi (2023)**	Y	Y	Y	CD	N	CD	Y	N	Y	Y	N	NA	Poor
**Inoue (2024)**	Y	Y	Y	CD	N	Y	Y	N	N	Y	N	NA	Poor (>20% attrition in a very small cohort)
**Angsuwatch. (2025)**	Y	Y	Y	CD	N	Y	N	N	CD	Y	N	NA	Poor

Distribution: Good = 1 (Hatch), Fair = 3 (Langton, Madsen, Soo), Poor = 10 (all others).

Table S3: Newcastle-Ottawa Scale (NOS) for Comparative Cohorts

Study (Author, Year)	Selection (Max 4★)	Comparability (Max 2★)	Outcome (Max 3★)	Total Stars (Max 9★)	Quality Rating
Menzella (2021)	★★★ (3/4)	— (0/2)	★★ (2/3)	5★	Poor / High risk of bias — confounding by indication not addressed (no statistical adjustment for significant baseline imbalance across the four treatment arms)

Figure S2: Sensitivity analysis for AQLQ in real-world studies (change from baseline). (A) Pooled estimates under alternative assumptions; random-effects model (REML) unless stated. (B) Leave-one-out analysis. CI, confidence interval; PI, prediction interval; REML, restricted maximum likelihood.

A)

**Analysis**	**k**	**n**	**Mean change (95% CI)**	**95% PI**	**I²**
Primary analysis (REML, r = 0.5)	11	267	1.38 (0.97 to 1.80)	−0.13 to 2.89	88.0%
Hartung–Knapp–Sidik–Jonkman adjustment	11	267	1.38 (0.91 to 1.85)	−0.13 to 2.89	88.0%
Pre–post correlation r = 0.3	11	267	1.39 (0.98 to 1.80)	−0.07 to 2.85	83.5%
Pre–post correlation r = 0.7	11	267	1.37 (0.96 to 1.79)	−0.18 to 2.93	92.5%
Excluding Torrego et al. (overlap)	10	211	1.41 (0.95 to 1.87)	−0.22 to 3.04	88.6%
Excluding Burn et al. (overlap)	10	239	1.45 (1.01 to 1.88)	−0.10 to 2.99	88.0%
Excluding Torrego et al. and Menzella et al. (overlap)	9	181	1.43 (0.92 to 1.95)	−0.35 to 3.21	89.5%
Excluding mini-AQLQ studies (Seeley, Madsen)	9	226	1.30 (0.82 to 1.78)	−0.39 to 2.99	90.1%
Excluding converted data (Madsen, Angsuwatcharakorn)	9	234	1.38 (0.87 to 1.89)	−0.40 to 3.15	90.2%
Leave-one-out (range)	10	—	1.23 to 1.50 (all 95% CIs excluding 0)	—	65.2–89.1%

B)

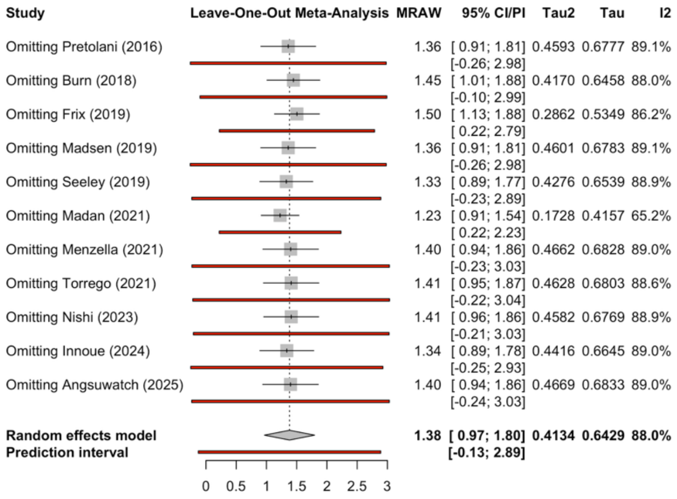

Figure S3: Sensitivity analyses for ACQ in real-world studies (change from baseline). (A) Pooled estimates under alternative assumptions; random-effects model (REML) unless stated. (B) Leave-one-out analysis. CI, confidence interval; PI, prediction interval; REML, restricted maximum likelihood.

A)

**Analysis**	**k**	**n**	**Mean change (95% CI)**	**95% PI**	**I²**
Primary analysis (REML, r = 0.5)	7	195	−1.24 (−1.86 to −0.62)	−3.37 to 0.89	94.5%
Hartung–Knapp–Sidik–Jonkman adjustment	7	195	−1.24 (−2.01 to −0.47)	−3.37 to 0.89	94.5%
Pre–post correlation r = 0.3	7	195	−1.25 (−1.87 to −0.62)	−3.37 to 0.87	93.1%
Pre–post correlation r = 0.7	7	195	−1.23 (−1.85 to −0.62)	−3.37 to 0.90	95.9%
Hatch et al. replacing Langton et al. (overlap)	7	195	−1.23 (−1.84 to −0.61)	−3.34 to 0.88	94.4%
Excluding converted data (Madsen, Madan)	5	143	−0.94 (−1.40 to −0.48)	−2.43 to 0.56	85.2%
Leave-one-out (range)	6	—	−1.39 to −0.99	—	81.7–95.4%

B)

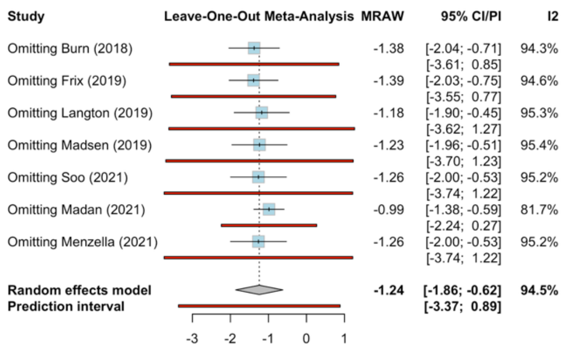

Figure S4: Sensitivity analyses for annual exacerbation rate in real-world studies (change from baseline, exacerbations per patient-year). (A) Pooled estimates under alternative assumptions; random-effects model (REML) unless stated. (B) Leave-one-out analysis. CI, confidence interval; PI, prediction interval; REML, restricted maximum likelihood.

A)

**Analysis**	**k**	**n**	**Mean change (95% CI)**	**95% PI**	**I²**
Primary analysis (REML, r = 0.5)	5	155	−3.79 (−6.98 to −0.61)	−14.62 to 7.04	95.3%
Hartung–Knapp–Sidik–Jonkman adjustment	5	155	−3.79 (−8.35 to 0.76)	−14.62 to 7.04	95.3%
Pre–post correlation r = 0.3	5	155	−3.79 (−6.97 to −0.61)	−14.53 to 6.95	94.1%
Pre–post correlation r = 0.7	5	155	−3.79 (−6.99 to −0.60)	−14.71 to 7.13	96.5%
Hatch et al. replacing Langton et al. (overlap)	5	155	−3.32 (−6.34 to −0.31)	−13.60 to 6.95	94.6%
Adding Madan et al. and Nishi et al. (converted data)	7	212	−3.32 (−5.63 to −1.00)	−11.26 to 4.63	93.7%
Leave-one-out (range)	4	—	−4.67 to −2.57 (3 of 5 95% CIs including 0)	—	92.9–96.1%

B)

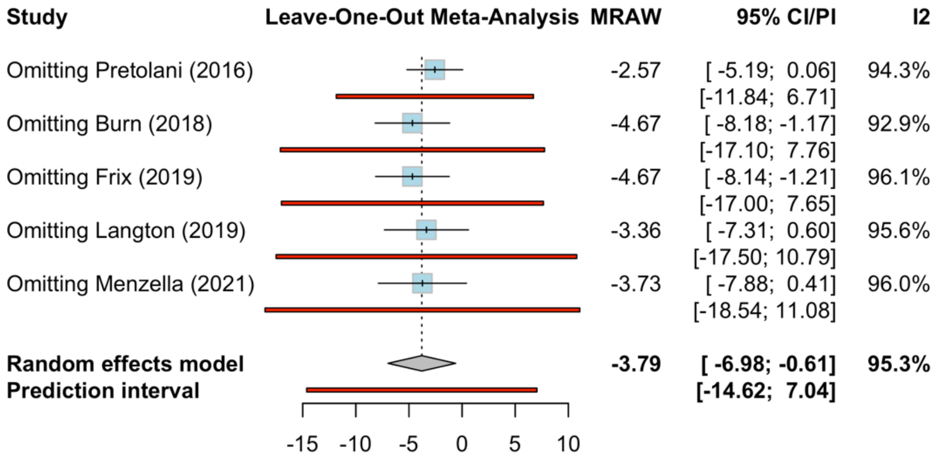

Table S4: Data extraction for the RCT meta-analysis of asthma control (ACQ).
Change from baseline in ACQ score in the BT and control arms, with source location, statistic as reported, timepoint, transformation applied, value entered and value previously entered.

Study	Source location	Statistic as reported (BT vs control)	Timepoint	n analysed (BT/C)	Transformation	Value entered (BT vs control)
Castro 2010	Table 2, PDF p5	Mean change −0.82 ± 0.95 vs −0.77 ± 1.08	12 mo	190/98	None	−0.82 ± 0.95 vs −0.77 ± 1.08
Cox 2007	Abstract, PDF p1; n from Figure 1, PDF p3	Reduction 1.2 ± 1.0 vs 0.5 ± 1.0	12 mo	49/46	Sign set negative (reduction)	−1.20 ± 1.00 vs −0.50 ± 1.00
Goorsenberg 2021	Table 2, PDF p6	Median change −0.79 (IQR −1.61 to 0.02) vs 0.09 (IQR −0.25 to 1.17)	6 mo (control: 6 mo standard care)	18/20	Median/IQR → mean/SD (Wan)	−0.79 ± 1.31 vs 0.351- ± 1.13
Leroux 2024	Table 2 and section 3.4.2, PDF p4	Mean change −0.99 (95% CI −1.67 to −0.30) vs −0.003 (95% CI −0.89 to 0.89)	V1→V5 (15 mo)	13/10	95% CI → SD (t, df 12 and 9)	−0.99 ± 1.13 vs −0.003 ± 1.24
Pavord 2007	Results, reduced-steroid phase, PDF p5	Mean change −0.99 ± 0.83 vs −0.22 ± 0.78 (P = 0.01)	52 wk	15/17	None	−0.99 ± 0.83 vs −0.22 ± 0.78

Table S5: Data extraction for the RCT meta-analysis of asthma-related quality of life (AQLQ). Change from baseline in AQLQ score (higher scores indicate better quality of life) in the bronchial thermoplasty (BT) and control arms of each trial, with source location, statistic as reported, timepoint, transformation applied, value entered in the meta-analysis and value entered in the original submission.

Study	Source location	Statistic as reported (BT vs control)	Timepoint	n analysed (BT/C)	Transformation	Value entered (BT vs control)
Castro 2010	Table 2, PDF p5	Mean change 1.35 ± 1.10 vs 1.16 ± 1.23	Integrated: mean of 6, 9, 12 mo (Table 2 footnote *)	190/98	None	1.35 ± 1.10 vs 1.16 ± 1.23
Cox 2007	Abstract, PDF p1; n from Figure 1, PDF p3	Mean change 1.3 ± 1.0 vs 0.6 ± 1.1	12 mo	49/46	None	1.30 ± 1.00 vs 0.60 ± 1.10
Goorsenberg 2021	Table 2, PDF p6	Median change 0.83 (IQR −0.15 to 1.69) vs −0.02 (IQR −0.77 to 0.75)	6 mo (control: 6 mo standard care)	18/20	Median/IQR → mean/SD (Wan)	0.79 ± 1.48 vs −0.01 ± 1.21
Leroux 2024	Table 2 and section 3.4.4, PDF p4	Mean change +1.19 (95% CI 0.64 to 1.75) vs +0.24 (95% CI −0.32 to 0.80)	V1→V5 (15 mo)	13/10	95% CI → SD (t, df 12 and 9)	1.19 ± 0.92 vs 0.24 ± 0.78
Pavord 2007	Results, reduced-steroid phase, PDF p4	Mean change 1.53 ± 0.79 vs 0.42 ± 0.82 (P = 0.001)	52 wk	15/17	None	1.53 ± 0.79 vs 0.42 ± 0.82

Table S6: Data extraction for the RCT meta-analysis of morning peak expiratory flow (PEF). Change from baseline in morning PEF (L/min; higher values indicate improvement) in the BT and control arms, with source location, statistic as reported, timepoint, transformation applied, and value entered.

Study	Source location	Statistic as reported (BT vs control)	Timepoint	n analysed (BT/C)	Transformation	Value entered (BT vs control)
Castro 2010	Table 2, PDF p5	Baseline 383.8 ± 104.32 → 411.6 ± 110.45 vs baseline 386.3 ± 112.59 → 408.7 ± 117.56	12 mo	190/98	Change = follow-up − baseline; SD of change imputed	27.8 ± 107.52 vs 22.4 ± 115.16
Cox 2007	Abstract, PDF p1; n from Figure 1, PDF p3	Mean change 39.3 ± 48.7 vs 8.5 ± 44.2	12 mo	49/46	None	39.3 ± 48.7 vs 8.5 ± 44.2
Pavord 2007	Baseline: Table 1, PDF p3; Figure	Baseline 355.9 ± 93.4 vs 350.4 ± 91.0; follow-up digitised	[22/52 wk]	15/17	Follow-up digitised (WebPlotDigitizer); change = follow-up − baseline; SD from error bars [SD/SE × √n]	24.4 ± 53.1 vs 8.7 ± 39.1

Table S7: Data extraction for the RCT meta-analysis of rescue medication use. Change from baseline in rescue short-acting β_2-agonist use (puffs/week; negative values indicate reduced use) in the BT and control arms, with source location, statistic as reported, timepoint, transformation applied, and the value entered.

Study	Source location	Statistic as reported (BT vs control)	Timepoint	n (BT/C)	Transformation	Value entered
Castro 2010	Table 2, PDF p5	13.4 ± 19.17 → 7.4 ± 15.01 vs 11.8 ± 11.24 → 7.5 ± 12.60	12 mo	190/98	Change; SD imputed, r = 0.5	−6.0 ± 17.47 vs −4.3 ± 11.98
Cox 2007	Online Supplement, PDF p12; n from Figure 1, PDF p3	19.8 ± 17.2 → 10.9 ± 15.0 vs 16.0 ± 18.8 → 14.8 ± 21.2	12 mo	49/46	Change; SD imputed, r = 0.5	−8.9 ± 16.21 vs −1.2 ± 20.11
Pavord 2007	Results, reduced-steroid phase, PDF p4	Change −25.6 ± 31.2 vs −6.1 ± 12.4	52 wk	15/17	None	−25.6 ± 31.2 vs −6.1 ± 12.4

Table S8: Data extraction for the RCT meta-analysis of symptom-free days. Change from baseline in the percentage of symptom-free days (higher values indicate improvement) in the BT and control arms, with source location, statistic as reported, timepoint, transformation applied, and value entered.

Study	Source location	Statistic as reported (BT vs control)	Timepoint	n (BT/C)	Transformation	Value entered
Castro 2010	Table 2, PDF p5	16.4 ± 24.04 → 40.8 ± 38.22 vs 16.8 ± 23.10 → 37.9 ± 36.95	12 mo	190/98	Change; SD imputed, r = 0.5	24.4 ± 33.46 vs 21.1 ± 32.33
Cox 2007	Abstract, PDF p1; n from Figure 1, PDF p3	Mean change 40.6 ± 39.7 vs 17.0 ± 37.9	12 mo	49/46	None	40.6 ± 39.7 vs 17.0 ± 37.9
Pavord 2007	Baseline: Table 1, PDF p3; -figure	Baseline 5.2 ± 13.6 vs 13.6 ± 24.3; follow-up digitised	[22/52 wk]	15/17	WebPlotDigitizer; SD	21.6 ± 30.6 vs 17.9 ± 43.4

Table S9: Data extraction for the RCT meta-analysis of hospitalisation for respiratory events. Number of participants with at least one hospitalisation for respiratory events, divided by the number of participants, in the BT and control arms, reported separately for the treatment period (up to 6 weeks after the final bronchoscopy) and the post-treatment period. Each participant is counted once per period, and periods were analysed separately.

Study	Period	Source location	Reported	n (BT/C)	Value entered
Castro 2010	Treatment	Results, PDF p5	16 subjects (8.4%) vs 2 (2.0%)	190/98	16/190 vs 2/98
Castro 2010	Post-treatment	Results, PDF p5	5 subjects (2.6%) vs 4 (4.1%)	190/98	5/190 vs 4/98
Cox 2007	Treatment	Results, PDF p7; n from Table 1, PDF p6	4 subjects vs 2	55/54	4/55 vs 2/54
Cox 2007	Post-treatment	Results, PDF p7	3 subjects vs 2	55/54	3/55 vs 2/54
Pavord 2007	Treatment	Abstract, PDF p1; Table 3, PDF p4	4 of 15 vs 0 of 17	15/17	4/15 vs 0/17
Pavord 2007	Post-treatment	Results and Table 3, PDF p4	3 subjects vs 1	15/17	3/15 vs 1/17

Table S10: Data extraction for the single-arm meta-analysis of asthma-related quality of life (AQLQ) in real-world studies

Study	Source location	Statistic as reported	Timepoint	n	Transformation	Value entered (mean ± SD of change)
Pretolani 2017	Table I, p. 3	2.6 ± 0.9 → 4.2 ± 1.5 (mean ± SD)	12 months	15	SD of change imputed (r = 0.5)	1.60 ± 1.31
Burn 2019	Table 2, p. 5	Paired mean change 0.75 (n = 28); baseline 3.64 (1.26), 12 months 4.24 (1.45)	12 months	28	Reported change; SD of change imputed (r = 0.5)	0.75 ± 1.36
Frix 2019	Tableau II, p. 4	2.9 (1.0) → 2.9 (1.0), N = 8 at 12 months (mean (SD))	12 months	8	SD of change imputed (r = 0.5)	0.00 ± 1.00
Madsen 2019	Table 2, p. 12	Median (IQR) 4.0 (3.2–4.9) → 5.5 (5.0–6.4)	12 months	16	Median/IQR → mean/SD (Wan); SD of change imputed (r = 0.5)	1.60 ± 1.28
Seeley 2019	Results and Table 1, p. 3; completion, p. 2	3.6 ± 0.3 → 5.6 ± 0.3; change 2.0 ± 0.4; baseline SD 1.3 (Table 1)	12 months	25	± interpreted as SEM; SD = SEM × √n	2.00 ± 2.00
Madan 2021	Table 3, p. 4	2.89 ± 1.18 → 5.59 ± 0.96 (mean ± SD)	Post-BT (not reported)	36	SD of change imputed (r = 0.5)	2.70 ± 1.09
Menzella 2021	Table 5, p. 11	3.6 ± 1.1 → 4.8 ± 1.2 (mean ± SD)	12 months	30	SD of change imputed (r = 0.5)	1.20 ± 1.15
Nishi 2023	Results, p. 2; Table 1	4.3 ± 1.2 → 5.4 ± 1.2 (mean ± SD)	12 months	21	SD of change imputed (r = 0.5)	1.10 ± 1.20
Inoue 2024	Results, p. 3	3.43 ± 1.21 → 5.28 ± 1.3	6 months	15	SD of change imputed (r = 0.5)	1.85 ± 1.26
Torrego 2021	Results, p. 4	3.26 ± 1.10 (n = 148) → 4.39 ± 1.50 (mean ± SD)	24 months	56	SD of change imputed (r = 0.5)	1.13 ± 1.35
Angsuwatcharakorn 2025	Table 2, p. 5	Median change 1.22 [IQR width 1.11]	12 months	17	Median used as mean; SD = IQR width / Wan factor (quartiles not reported)	1.22 ± 0.90
Soo 2021	Results, p. 2; Table 2	Median (IQR) 3.4 (2.5–3.6) → 4.3 (3.9–4.6)	12 months	17	—	Not included

Table S11: Data extraction for the single-arm meta-analysis of asthma control questionnaire (ACQ) in real-world studies

Study	Source location	Statistic as reported	Timepoint	n	Transformation	Value entered (mean ± SD of change)
Burn 2019	Table 2, p. 5	Paired mean change −0.43 (n = 36); baseline 3.28 (1.36), n = 49; 12 months 2.75 (1.34), n = 40	12 months	36	Reported change; SD of change imputed (r = 0.5)	−0.43 ± 1.35
Frix 2019	Tableau II, p. 4	3.6 (0.9) → 3.3 (0.8), N = 9 at 12 months (mean (SD))	12 months	9	SD of change imputed (r = 0.5)	−0.30 ± 0.85
Langton 2020	Table 2, p. 23; n, p. 9	3.2 ± 1.0 → 1.6 ± 1.2 (mean ± SD)	12 months	51	SD of change imputed (r = 0.5)	−1.60 ± 1.11
Madsen 2019	Table 2, p. 12	Median (IQR) 2.9 (2.1–3.7) → 1.4 (0.9–2.6)	12 months	16	Median/IQR → mean/SD (Luo; Wan); SD of change imputed (r = 0.5)	−1.25 ± 1.34
Soo 2024	Table 2, p. 2	3.7 ± 0.5 → 2.6 ± 0.8 (mean ± SD)	12 months	17	SD of change imputed (r = 0.5)	−1.10 ± 0.70
Madan 2021	Table 3, p. 4	Median (IQR) 3.91 (3.1–4.6) → 1.08 (0.83–1.3)	Post-BT (not reported)	36	Median/IQR → mean/SD (Luo; Wan); SD of change imputed (r = 0.5)	−2.80 ± 1.03
Menzella 2021	Table 5, p. 11	3.2 ± 0.9 → 2.1 ± 0.9 (mean ± SD)	12 months	30	SD of change imputed (r = 0.5)	−1.10 ± 0.90
Hatch 2023	Abstract and Results	3.0 ± 1.0 → 1.8 ± 1.0 at 5 years	60 months	51	—	Not included
Hara 2023	Figure	ACQ-6 change shown graphically only	24 months	10	—	Not included

Table S12: Data extraction for the single-arm meta-analysis of exacerbation rate/year

Study	Source location	Statistic as reported	Timepoint	n	Transformation	Value entered (mean ± SD of change)
Pretolani 2017	Table I, p. 3 (footnote: annual rates as mean ± SE)	Annual rate of severe exacerbations 9.7 ± 1.3 → 0.7 ± 0.4 (mean ± SE)	12 months	15	SE → SD (× √n); SD of change imputed (r = 0.5)	−9.00 ± 4.47
Burn 2019	Table 2, p. 5	Rescue steroid courses (annualised), median [LQ–UQ]: 4 [2–5.5] (n = 75) → 3.1 [1.4–5.8] (n = 55); paired comparison n = 49	12 months	49	Median/IQR → mean/SD (Luo; Wan); SD of change imputed (r = 0.5)	−0.37 ± 3.06
Frix 2019	Tableau II, p. 4	Exacerbations in previous year 2.9 (2.8) → 2.7 (2.6) (mean (SD))	12 months	10	SD of change imputed (r = 0.5)	−0.20 ± 2.71
Langton 2020	Table 2, p. 23; n, p. 9	Exacerbations per 6 months 3.7 ± 3.3 → 0.9 ± 1.5 (mean ± SD)	12 months	51	Annualised (× 2 for mean and SD); SD of change imputed (r = 0.5)	−5.60 ± 5.72

Menzella 2021	Table 5, p. 11	Exacerbations in last year 5.6 ± 3.4 → 1.5 ± 1.0 (mean ± SD)	12 months	30	SD of change imputed (r = 0.5)	−4.10 ± 3.03
Hatch 2023	Table 2, p. 4	Exacerbations per annum 3.9 ± 3.6 → 0.6 ± 1.0 (mean ± SD)	12 months	51	SD of change imputed (r = 0.5)	Sensitivity analysis only (−3.30 ± 3.22)
Madan 2021	Table 3, p. 4	Exacerbations per year, median (IQR): 3 (1–10) → 0.5 (0–3)	Post-BT (not reported)	36	—	Not included
Nishi 2023	Results, p. 2	Exacerbations per year, median (IQR): 1.0 (0.5–3.5) → 0.0 (0.0–2.0)	12 months	21	—	Not included
Angsuwatcharakorn 2025	Table 4, p. 6	Systemic corticosteroid bursts per patient-year, median [IQR width]: 4 [10] → 1 [4]	12 months	17	—	Not included
